# Identification of Sodium/Myo‐Inositol Transporter 1 as a Major Determinant of Arterial Contractility

**DOI:** 10.1096/fj.202600244R

**Published:** 2026-09-11

**Authors:** Elizabeth A. Forrester, Skye Lau, Miguel Benitez‐Angeles, Christopher J. Garland, Vincenzo Barrese, Anthony P. Albert, Iain A. Greenwood

**Affiliations:** ^1^ School of Health and Medical Sciences City St George's, University of London London UK; ^2^ Department of Pharmacology University of Oxford Oxford UK; ^3^ Deptartment of Neuroscience, Reproductive Sciences and Dentistry University of Naples Federico II Naples Italy; ^4^ University of East Anglia Norwich UK

**Keywords:** arterial contractility, blood pressure regulation, membrane hyperpolarization, potassium channels, sodium/myo‐inositol transporter (SMIT1), vascular smooth muscle

## Abstract

As the sodium/myo‐inositol transporter (SMIT1) is a positive regulator of Kv7.4/7.5 channels in arterial smooth muscle, we postulated that altering SMIT1 expression could have a major impact upon vascular reactivity. Consequently, this study aimed to characterize the effects of changes of SMIT1 membrane abundance on vascular tone in 2nd order mesenteric arteries and left anterior coronary arteries from WistarHan rats and identify the molecular mechanisms involved. Morpholino‐mediated knockdown of SMIT1 enhanced U46619‐ and methoxamine‐mediated contractions of mesenteric artery while impairing relaxations to the Kv7 activator ML213, isoprenaline and Calcitonin Gene Related Peptide (CGRP). Conversely, augmenting SMIT1 membrane abundance by raising external osmolarity with 150 mM raffinose impaired receptor‐mediated contractions of mesenteric and coronary arteries, hyperpolarized the membrane potential and augmented relaxations to ML213 and adenosine. 1 h incubation in raffinose increased heterologously expressed Kv7.4‐generated K^+^ currents in a SMIT1‐dependent manner. Proximity ligation assays revealed that raffinose incubation increased the association of SMIT‐Kv7.4/7.5 as well as Kv7.4 with Gβγ subunits. The SGK1 inhibitor EMD638683 prevented the raffinose‐induced increase in SMIT1 and the anti‐contractile effect. Toggling the membrane abundance of SMIT1 had a dramatic effect on the arterial response to vasoconstrictors and vasodilators mediated by greater coordination of Kv7 channels and Gβγ subunits. This work identified SMIT1‐Kv7 channel complexes and SGK1 regulation as key modulators of arterial responsiveness.

## Graphical abstract

The tonicity‐responsive enhancer binding‐protein (TonEBP) and serum glucocorticoid kinase (SGK) sense a rise in extracellular hypertonicity resulting in an increase SMIT1 membrane abundance in vascular smooth muscle cells. SMIT1 associates with Kv7.4/5, Gβγ, and KCNE4, promoting hyperpolarization, reduced voltage‐gated calcium channel (VGCC) opening, and vasorelaxation. These findings highlight SMIT1 as a key regulator of arterial tone in resistance arteries. Tonicity‐responsive enhancer (TonE), Myo‐inositol (MI), phosphatidylinositol 4,5‐bisphosphate (PIP2). (Source: Author using Biorender.com).

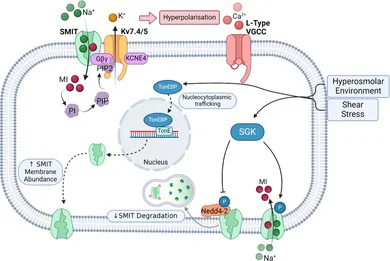

## 1 Introduction

The sodium/myo‐inositol transporter (SMIT1) encoded by Slc5a3 provides myo‐inositol required for the synthesis of the phospholipid phosphatidylinositol bisphosphate (PIP_2_), which has an important role in cellular signaling and is a crucial cofactor for many ion channels [1]. Beyond its transport function, SMIT1 has been identified to alter the ion selectivity, gating, and pharmacology of KCNQ‐encoded voltage‐gated potassium channels (Kv7.1–7.5) independent of inositol transport through a direct physical interaction with the channel pore component [2, 3, 4, 5, 6, 7]. These channel‐transporter complexes (‘chansporters’) have been implicated as important regulators of neuronal activity at rest. Moreover, chansporter complexes provide a mechanism linking cellular responses to increased external osmolarity [8, 9, 10]. Expression of Slc5a3 is enhanced by a tonicity‐responsive enhancer binding protein (TonEBP, [11]), while SMIT1 protein cell‐membrane abundance are influenced by ubiquitin ligases that are themselves downregulated by hypertonically‐regulated serum‐glucocorticoid sensitive kinase (SGK) 1 [10].

In arterial smooth muscle, activation of Kv7 channels prohibits arterial contraction and contributes to receptor‐mediated vasodilation [12, 13, 14]. SMIT1 associates with Kv7.4 and Kv7.5 in renal arterial smooth muscle cells, and overnight incubation in hypertonic medium markedly increased SMIT1 expression, augmented Kv7 channel currents, and reduced vasoconstrictor responses [6]. The juxtaposition of SMIT1 regulation by hypertonicity and a role as a large auxiliary channel for Kv7 channels offers an effective mechanism to transduce changes in external environmental status to arterial responses. We hypothesized that altering SMIT1 membrane abundance would modulate responses to receptor‐mediated dilators and offered a mechanism to fine‐tune arterial contractility. The present study provides clear evidence that SMIT1 is a key regulator of vascular smooth muscle function, advancing our mechanistic understanding of the molecular complexes that underpin arterial tone and contractility.

## 2 Methods

### 2.1 Animals

Experiments were performed on second‐order mesenteric and cardiac septal resistance arteries from male and female rats aged 11–14 weeks and weighing 175–300 g. Animals were maintained under an institutional site license and sacrificed by a Schedule 1 method (cervical dislocation) in accordance with the UK Animal (Scientific Procedures) Act 1986; therefore, no approval from a local or university ethics review board was required. This investigation conforms to Directive 2010/63/EU of the European Parliament on the protection of animals used for scientific purposes.

### 2.2 Wire Myography

Arteries were cut into ∼2 mm segments and mounted on 40 μm stainless steel wires in a myograph (DMT, Aarhus, Denmark). The myograph chambers contained physiological salt solution comprising of 119 mM NaCl, 4.5 mM KCl, 1.17 mM MgSO_4_.7H20, 1.18 mM NaH_2_PO_4_, 25 mM NaHCO_3_, 5 mM glucose, 1.25 mM CaCl_2_ that was bubbled with 95% oxygen and 5% carbon dioxide at 37°C. All vessels were subject to a normalization procedure to standardize the experimental conditions [15] and arteries were set to a internal circumference 90% of the diameter at in vivo transmural pressure (90 and 80 mmHg for mesenteric and coronary arteries respectively). Tension in each segment was first amplified by a PowerLab (ADInstruments, Oxford, UK) and then recorded using LabChart 8 Pro Software (RRID:SCR_017551; ADInstruments, Oxford, UK). Vessels were determined viable by first challenging with isotonic high K^+^ physiological salt solution (K^+^PSS) of the following composition 63.5 mM NaCl, 60 mM KCl, 1.17 mM MgSO_4_·7H_2_0, 1.18 mM NaH_2_PO_4_, 25 mM NaHCO_3_, 5 mM glucose, 1.25 mM CaCl_2_ and endothelial cell integrity was determined via a vasorelaxation in response to 10 μM of the synthetic acetylcholine analog, carbachol when each vessel was preconstricted with 10 μmol·L^−1^ methoxamine, an α_1_‐adrenoreceptor agonist. To raise external osmolarity, arteries were incubated for various durations (10 min to 6 h) in PSS containing 150 mM of the non‐metabolizable sugar raffinose used previously (reference [3]) to characterize SMIT1 in neurons and rat renal arteries. 150 mM mannitol was also used.

### 2.3 Membrane Potential Recording

Segments of mesenteric artery were mounted on 25 μm gold‐plated tungsten wires in a myograph. Simultaneous measurement of isometric tension and vascular smooth muscle cell membrane potential (*V*_m_) was recorded in mesenteric arteries bathed in normal physiological salt solution or one supplemented with 150 mM raffinose in the presence and absence of pan Kv7 blocker XE991 (10 μM). Membrane potentials were recorded through a preamplifier (Neurolog system, Digitimer Ltd., Welwyn Garden City, UK) linked to a MacLab data acquisition system (model 4e, AD Instruments) and LabChart software (v8.1.17 ad Instruments). Individual vascular smooth muscle cells were impaled with sharp glass microelectrodes (backfilled with 2 M KCl; tip resistances approximately 60 MΩ), observed as a rapid deflection toward the resting membrane potential, near −50 mV as shown in [16].

### 2.4 Single Cell Electrophysiology

HEK293 cells were cultured in DMEM supplemented with fetal bovine serum, seeded on glass coverslips, and transfected with Kv7.4 channel pcDNA alone or co‐transfected with SMIT1 pcDNA at a 1:1 ratio using Lipofectamine 2000 reagent (Thermo Fisher Scientific). After 24 h, K^+^ currents generated by Kv7.4 expression alone and with SMIT1 currents were recorded in the whole‐cell configuration of the patch‐clamp technique using a voltage‐step protocol from a holding potential of −60 mV, with 10 mV increments from −70 to +40 mV applied for 750 ms, after which the voltage was returned to −40 mV for 125 ms. Signals were amplified using an Axopatch 200B amplifier and a Digidata 1322A (Axon Instruments) sampled at 10 kHz, and low‐pass filtered at 2 kHz. The patch pipettes were pulled from borosilicate glass with a resistance of 4–6 megohms. The bath solution contained (mM): 140 NaCl, 4 KCl, 1 CaCl_2_, 1 MgCl_2_, 10 Glucose and 10 HEPES pH 7.2; and the pipette solution contained (mM): 130 KCl, 5 BAPTA, 10 HEPES, 5 K_2_ATP, 1 MgATP, pH 7.3. Coverslips with Kv7.4 and Kv7.4 + SMIT1 expressing cells were incubated for 1 h in DMEM supplemented with raffinose (150 mM; Sigma Aldrich).

Data were acquired, analyzed and plotted using Clampex, Clampfit (Molecular Devices) and Igor Pro (Wavemetrics Inc.). Currents at each voltage were normalized to cell capacitance to obtain current density (pA/pF); conductance was calculated from tail currents and normalized to the maximum value and a Boltzmann distribution was fitted to calculate the *V*½. All data are expressed as mean ± SEM. Statistical analysis was performed using JASP (Version 0.96.0). Current density–voltage and conductance–voltage relationships were analyzed using two‐way ANOVA followed by Bonferroni post hoc test. A *p* < 0.05 was considered statistically significant.

### 2.5 Morpholino Transfection and Immunohistochemistry

Knockdown of SMIT1 in mesenteric arteries was performed by transfection with specific morpholino oligonucleotides (See [6, 17, 18]), targeting mRNA from 5′ cap of the untranslated region (UTR) and the following 25 bases of the amino acid coding sequence to inhibit translation however does not affect transcript levels. (5′ GTCTCCAGCACAGCCCTCATCTG 3′). The morpholino used in the present study to reduce SMIT1 levels was assessed and verified by immunocytochemistry in [6] and in the present study. Morpholinos were obtained from Gene Tools Inc., Philomath, OR, USA (RRID: SCR_005663) and came lyophilized at a concentration of 300 nM for target‐knockdown morpholinos and 100 nM for scrambled control morpholinos. Morpholino nucleotides were mixed with Opti‐MEM reduced‐serum medium (Thermo Fisher Scientific, 31 985 062) and Lipofectamine RNAiMAX Transfection Reagent (Thermo Fisher Scientific, 13 778 075) and left at room temperature for 2 h. Morpholino/Lipofectamine/Opti‐MEM mixture was added to Dulbecco's modified Eagle medium (DMEM) F‐12 (Sigma, UK) containing 1% penicillin/streptomycin giving a final morpholino concentration of 10 μM. Arteries were then added to the mixture in the 24‐well plate and incubated at 37°C in 5% CO_2_ for 48–72 h. Knockdown morpholino samples were run in parallel with a scrambled oligo control with no target or activity. Knockdown of protein was assessed by immunohistochemistry following myography experiments.

Arterial segments were fixed in situ in myograph chambers with 4% paraformaldehyde (J61899, Thermo Scientific) for 1 h at room temperature. Arteries were then incubated for 90 min at room temperature with blocking buffer containing 1% bovine serum albumin (BSA), 0.5% Triton X‐100, 0.05% Tween 20 in phosphate buffered saline (PBS) and incubated overnight with primary anti‐mouse SMIT1 monoclonal antibody, CD31/PCAM1 and smooth muscle myosin heavy chain 11 conjugated antibodies shown in Table 2. This was followed by a secondary antibody incubation with donkey anti‐mouse secondary (Alexa Fluor 546, A10036, Invitrogen), the endothelial cell marker mouse/rat cd31/pecam‐1 alexa fluor 488‐conjugated antibody (AF3628‐SP, R & D systems, 1:150) and smooth muscle myosin heavy chain 11 alex flour 647‐conjugated antibody, all diluted in blocking buffer for 90 min at room temperature. Arteries were then placed in mounting medium (Vectashield Plus Antifade, Vector Laboratories) and laid flat between two glass coverslips. Whole arteries were imaged using a laser‐scanning confocal microscope (FV1200; Olympus) using a 40× water‐immersion objective (1.15 NA, 1024 × 1024 pixels; Olympus). Sequential z‐stacks were acquired at 0.5 μm intervals using Fluoview software (FV10‐ASW 3.0; Olympus) and analyzed for mean fluorescence intensity in Imaris software (v10.2.0; Bitplane).

### 2.6 Oestrus Cycle Stage Determination

A vaginal smear was taken following euthanasia, whereby 50 μL of physiological saline solution (PSS) was flushed 4–6 times into the vaginal canal using a 20–200 μL pipette tip to liberate cells from the cervix. The resulting cell suspension was placed onto a glass coverslip and examined under a light microscope (Olympus CK40). The relative abundance and morphology of the four principal cell types, together with uterine morphology, were used to classify each animal as being in proestrous, estrous, metestrous, or diestrous stages ([19, 20]). Serum sex hormone concentrations were not directly measured in the present study. However, we have previously quantified circulating estrogen and other sex hormones across the rat estrous cycle using targeted liquid chromatography–tandem mass spectrometry (LC–MS/MS) (see [21] for blood concentrations).

### 2.7 mRNA Extraction and Reverse Transcription Quantitative Polymerase Chain Reaction (RT‐qPCR)

mRNA from whole mesenteric arteries was extracted using Monarch Total RNA Miniprep Kit (New England Biolabs, Ipswich, Massachusetts, USA). Each RNA sample was reversed‐transcribed to a more stable complementary DNA (cDNA) sample using LunaScript RT Supermix (New England Biolabs, Ipswich, Massachusetts, USA) and a T100 thermal cycler (1 861 096, Bio‐Rad, Hercules, California, USA). Quantitative analysis of relative gene expression was determined via CFX‐96 Real‐Time PCR Detection System (RRID:SCR_018064, Bio‐Rad, Hertfordshire, UK).

Samples were run duplicate in a BrightWhite qPCR plate (Primer Design, Camberley, UK) in combination with PrecisionPLUS qPCR Master Mix (Primer Design, Camberley, UK), 300 nmol·L^−1^ of gene‐specific target primer (Thermo Fisher Scientific, Waltham, Massachusetts, USA) and 10‐ng cDNA as per manufacturers instruction. A no template control (NTC) and NRT sample were run for each gene and the quantification cycle (*C*_q_) was determined via Bio‐Rad CFX96 Manager 3.0. *C*_q_ values are expressed as normalized values to appropriate, stable, housekeeper genes (2^−Δ*Cq*^) GAPDH and cytochrome C1 (*Cyc1*) chosen for their stable, and similar *C*_q_ values. Housekeeper genes were acquired from Primer Design (Camberley, UK) and shown in Table 1.

**TABLE 1:** List of primers used for qPCR.

Primer	Forward sequence (5′ – 3′) Reverse sequence (5′ – 3′)	Amplicon (BP)	Accession number
Slc5a3	GCATTAAGGGGCTCCAACCT ATGAGAGCTGCTTGCCCATT	136	NM_053715.2
Slc5a11	TCCCTGGTATGGTGAGCAGA CTGTGGGCAAAAGTTCCAGC	141	XM_039101532.1
Gapdh	TGTTCTAGAGACAGCCGCAT AAATCCGTTCACACCGACCT	83	NM_017008.4
Cyc1	TGTTTTCCTTGCTCACTGGC TGGCAACATCCTTGGCTACT	183	NM_001277194.1

### 2.8 Immunocytochemistry

Vascular smooth muscle cells were isolated from whole mesenteric and coronary arteries via incubation in isolation solution comprised of 120 mM NaCl, 6 mM KCl, 12 mM glucose, 10 mM HEPES and 1.2 mM MgCl2 supplemented with 1.75 mg/mL Collagenase Type IA, 0.9 mg/mL protease, 1 mg/mL trypsin inhibitor and 1 mg/mL bovine serum albumin (Sigma, UK) at 37°C for 17 min. Vessels then underwent mechanical trituration by wide‐bore glass pipette to liberate vascular smooth muscle cells from the extracellular matrix. The subsequent cell suspension was plated onto 13‐mm coverslips in a 24‐well plate, supplemented with an equal volume of Ca^2+^ (2.5 mmol·L^−1^) containing PSS and left to attach for 1 h. Vascular smooth muscle cells were fixed in 4% paraformaldehyde solution diluted in filtered phosphate‐buffered saline (PBS) for 15 min and then stored at 4°C in PBS prior to staining.

The cells were incubated with 0.1 M Glycine solution diluted in PBS (5 min), blocking solution containing 0.1% Triton X100 + 0.5% Bovine Serum Albumin (BSA) diluted in PBS (45 min) and the following incubation with primary anti‐mouse SMIT1 monoclonal antibody (3A6: sc‐293 330, 1:200) diluted in blocking solution overnight at 4°C. The following day the cells were incubated in a secondary antibody (Donkey Anti‐ mouse, A21203, Alexa Fluor 594, 1:200, Invitrogen) diluted in blocking solution for 45 min. The coverslips were washed in PBS before being mounted onto a microscope slide with 4′,6‐diamidino‐2‐phenylindole (DAPI) (Vectashield, Vector Laboratories, Burlingame, CA) mounting medium and imaged using a Nikon A1 plus scanning Confocal Ti2 chassis microscope (Nikon Instruments Europe BV, Amsterdam, Netherlands). Excitation of the fluorophores was achieved with 405, 488, 536 and 635 nm lasers and images were acquired through a 60× oil immersion objective and corrected total cell fluorescence (CTCF) was analyzed using ImageJ (RRID:SCR_003070) software.

### 2.9 Proximity Ligation Assay

Proximity ligation assay (PLA) was used to assess the association in close proximity of SMIT1 with various ion channel proteins in isolated vascular smooth muscle cells from arteries incubated in either PSS or 150 mM raffinose for 4 h or overnight. The Duolink in situ PLA detection kit (Sigma Aldrich, Dorset, UK) was used according to the manufacturer's protocol. The cells were permeabilized in 0.1% triton X‐100 (5 min) and blocked for 30 min at 37°C with Duolink PLA blocking buffer (DUO82007, Sigma Aldrich, St. Louis, MO, USA). Cells were then incubated overnight at 4°C with a combination of two primary antibodies shown in Table 2 diluted in blocking buffer. The following day, cells were labeled with Duolink anti‐rabbit PLUS (DUO92002, Sigma Aldrich, St. Louis, MO, USA) and anti‐mouse MINUS (DOU92004, Sigma Aldrich, St. Louis, MO, USA) Duolink in situ oligonucleotide‐labeled probes for 1 h at 37°C (1:5). Hybridized oligonucleotides attached to the probes were ligated for 30 min at 37°C. Following this, cells were incubated for 100 min at 37°C in a solution containing an amplification buffer (DUO82011, 5×, Sigma Aldrich, St. Louis, MO, USA) containing red fluorophore oligonucleotide probes (568 nm) and polymerase (10 units/μL, 80×, DUO82030) to enable rolling circle amplification. The cells were then mounted on cover slides with a DAPI‐containing mounting medium (Vectashield, Vector Laboratories, Burlingame, CA) and then Z‐stack images were acquired using a Nikon A1R confocal microscope (Nikon Instruments Europe BV, Amsterdam, Netherlands) on Ti2 chassis (image resource facility, City St George's University of London). Analysis of mid‐cell *xy* sections were performed using Image‐J software.

**TABLE 2:** List of antibodies used for molecular experiments.

Antibody	#CATalog Source	Predicted MW, kDa	Raised in	Epitope	[Used]
Anti‐SMIT antibody (3A6)	sc‐293 330, Santa Cruz, Texas USA	~79	Mouse monoclonal	Raised against amino acids 533–641 human	1:100 [1 μg/mL]
Kv7.4/KCNQ4 antibody	APC‐164, Alomone Labs, Jerusalem, Israel	77	Rabbit polyclonal	Intracellular, C‐terminus of KCNQ4	1:200 [4 μg/mL]
Kv7.5/KCNQ5 antibody	ABN1372‐q2476155; Millipore, Temecula, CA, USA 1 mg/ml	~103	Rabbit	Human IgG	1:100 [10 μg/mL]
Gβ Antibody (M‐14)	sc‐261, 200 μg/mL, Santa Cruz, Texas USA	~45	Rabbit polyclonal	N‐terminus of Gβ1, IgG	1:200 [1 μg/mL]
Gβ Antibody (H‐1)	sc‐166 123, 200 μg/ml, Santa Cruz, Texas USA	~45	Mouse monoclonal	C‐terminus of G_β_ (amino acids 302–340)	1:200 [1 μg/mL]
CD31/PECAM1 Antibody ‐ conjugated to Alexa Fluor 488	FAB3628, R&D systems	~130 kDa	Goat Polyclonal	Extracellular domain of CD31 raised against Glu18‐Lys590	1:80
Smooth muscle myosin heavy chain 11 ‐conjugated to Alexa Fluor 647	ab196982, clone number EPR5336B, Abcam	~227 kDa	Rabbit Recombinant Monoclonal	Proprietary information	1:200 [2.5 μg/mL]
Serum/Glucocorticoid Regulated Kinase 1 antibody	orb129741	~49 kDa	Rabbit Polyclonal	Proprietary information	1:200 [5 μg/mL]

### 2.10 Data and Statistical Analysis

All values from functional experiments were expressed as mean ± standard error of the mean (SEM) with no less than 5 individual data points, each representing a biological repeat. For functional experiments, cumulative concentration effect curves were produced. The tone of the artery was recorded after each subsequent addition of a given pharmacological agent and the values were formulated as a percentage of the maximum contraction. Using GraphPad Prism (RRID:SCR_002798, Version 10.0.0) a transformed data set of mean values was generated using *X* = Log(*X*), to reduce representative skew. A four parametric linear regression analysis was then performed to produce a concentration effect curve on a log(*x*) graph with the standard error of mean (SEM). A two‐way ANOVA was performed followed by a post hoc Bonferroni test for multiple comparisons or one‐way ANOVA were used to determine statistical significance between groups, according to the different experiments. Significance values were represented as *p < 0.05, **p < 0.01, ***p < 0.001 and ****p < 0.0001.

All molecular biology experiments involved five biological repeats with a minimum of five cells recorded per sample and expressed mean ± SEM. When comparing multiple groups, a two‐way ANOVA was performed followed by a post hoc Bonferonni test. For data comparing two individual groups, a normality test under column analysis was performed to determine whether the data followed a Gaussian distribution. This involved performing a total of four normal distribution tests (D'Agostino and Pearson test, Anderson‐Darling test, Shapiro–Wilk test and Kolmogorov–Smirnov test) that calculate a *p* value. If the *p* value was greater than 0.05 it confirmed the data followed a Gaussian distribution and a two‐sample *t*‐test performed. If the *p* value was less than or equal to 0.05 the data did not follow a Gaussian distribution and a Mann Whitney test was performed. Significance values were represented as *p < 0.05, **p < 0.01, ***p < 0.001 and ****p < 0.0001. All data sets subject to statistical analysis met the minimum sample size requirement containing ≥ 5 biological repeats, where *N* = number of independent values and *n* = number of technical repeats.

## 3 Results

### 3.1 Downregulating SMIT1 Levels Increases Arterial Contractility and Impairs the Relaxation Responses to CGRP and Isoprenaline

**FIGURE 1:**
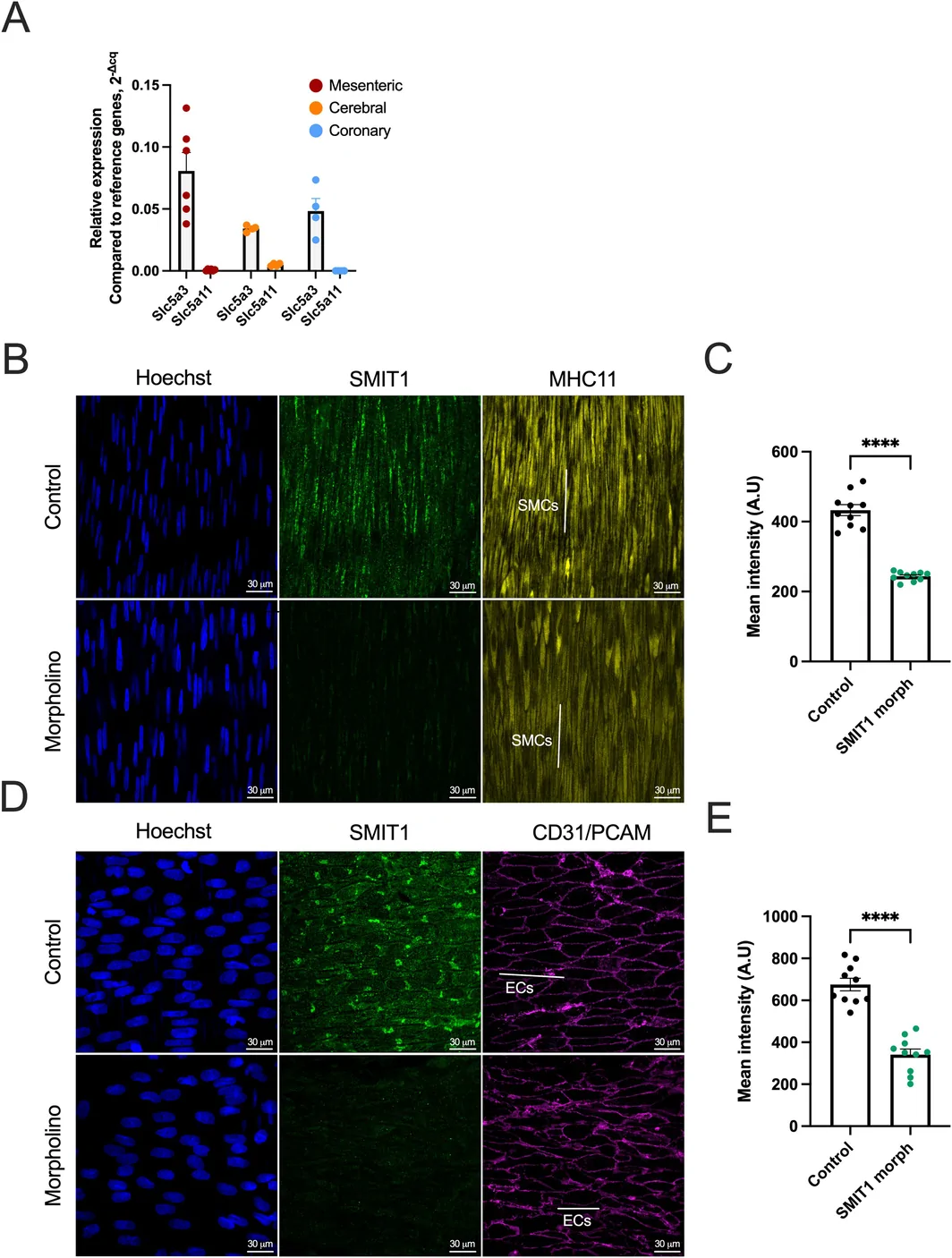
SMIT protein expression in control and knockdown vascular smooth muscle and endothelial cell layers of whole mesenteric arteries. The mean transcript abundance of Slc5a3 and Slc5a11 in mesenteric (red), coronary (blue) and cerebral (orange) arteries from male wister rats (A). The expression of SMIT1 protein (green) in the smooth muscle layer (B) and endothelial cell layer (D) of mesenteric arteries with Hoechst nuclear stain (blue), myosin heavy chain 11 (MHC11, yellow) and CD31/PCAM (purple). The mean fluorescence intensity of SMIT1 in smooth muscle (C) and endothelial cell layers (E) of mesenteric arteries transfected with scrambled control morpholino (black) and SMIT1 targeted morpholino (green). All data points are shown as mean ± SEM denoted by error bars, and statistical values are determined by a Mann–Whitney test where ****p < 0.0001.

**FIGURE 2:**
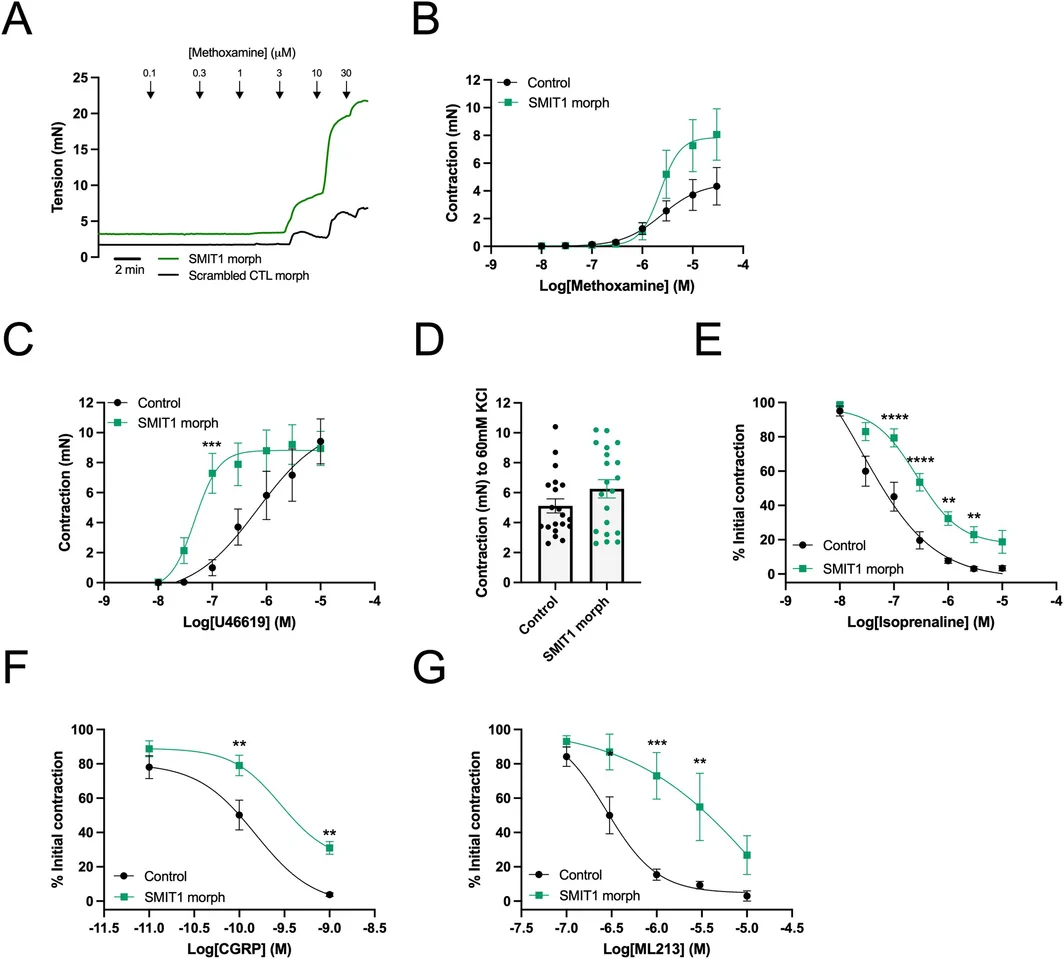
Effect of SMIT1 knock‐down on arterial contractile and relaxation responses. A representative trace of the concentration–effect curve to methoxamine in mesenteric arteries incubated with either a scrambled control morpholino (black) or a SMIT1‐targeting morpholino (green, A). The mean raw concentration‐dependent contraction (mN) to methoxamine (B) and U46619 (C) in mesenteric arteries incubated with either a scrambled control morpholino (black) or a SMIT1 targeted morpholino (green) (*N* = 8). The contraction to 60 mM KCl in mesenteric arteries incubated with either a scrambled control morpholino (black) or a SMIT1‐targeting morpholino (green, D, *N* = 20). The mean concentration‐dependent relaxation (mN) to Isoprenaline (E, 1 nM–1 μM), CGRP (F, 10 pM–1 nM) and ML213 (G, 100 nM–10 μM) in scrambled control morpholino (black) and SMIT1 targeted morpholino (green) mesenteric arteries pre‐contracted with 300 nM U46619 (*N* = 6). All data points are shown as mean ± SEM denoted by error bars, and statistical values are determined by 2‐way ANOVA with multiple comparisons are calculated using post hoc Bonferroni test where *p < 0.05, **p < 0.01, ***p < 0.001, ****p < 0.0001.

Quantitative PCR was undertaken to ascertain the relevant abundance of Slc5a3 and Slc5a11 that encode for SMIT1 and SMIT2. Figure 1A shows that mesenteric, cerebral and coronary arteries express Slc5a3 almost exclusively with negligible contribution from Slc5a11 (Figure 1A). To investigate the functional impact of SMIT1 in mesenteric arteries, we used morpholinos blocking the translation of SMIT1 shown by Barrese et al. [6] to reduce SMIT1 expression in single‐cells by roughly 50%. Whole artery immunohistochemistry revealed robust expression of SMIT1 in the smooth muscle (MHC11 positive) and endothelial (CD31/PCAM1 positive) layers of the mesenteric artery (Figure 1B–E), which was reduced markedly by transfection with a SMIT1‐specific morpholino (Figure 1C,E). Figure 2A–C shows that responses to methoxamine and U46619 were enhanced considerably by knockdown of SMIT1 whereas contractions to 60 mM KCl were not affected (Figure 2D). In addition, receptor‐mediated relaxation induced by isoprenaline and CGRP (Calcitonin gene related peptide) was impaired by SMIT1 knockdown (Figure 2E,F). Relaxations produced by 1 μM ML213 (Kv7.2–7.5 activator [21, 22]), were also reduced significantly by SMIT1 knockdown (*N* = 5, Figure 2G). These data highlight that reducing SMIT1 impacts vascular reactivity.

### 3.2 Acute Incubation of Hypertonic Raffinose Enhances Arterial Relaxation Responses

**FIGURE 3:**
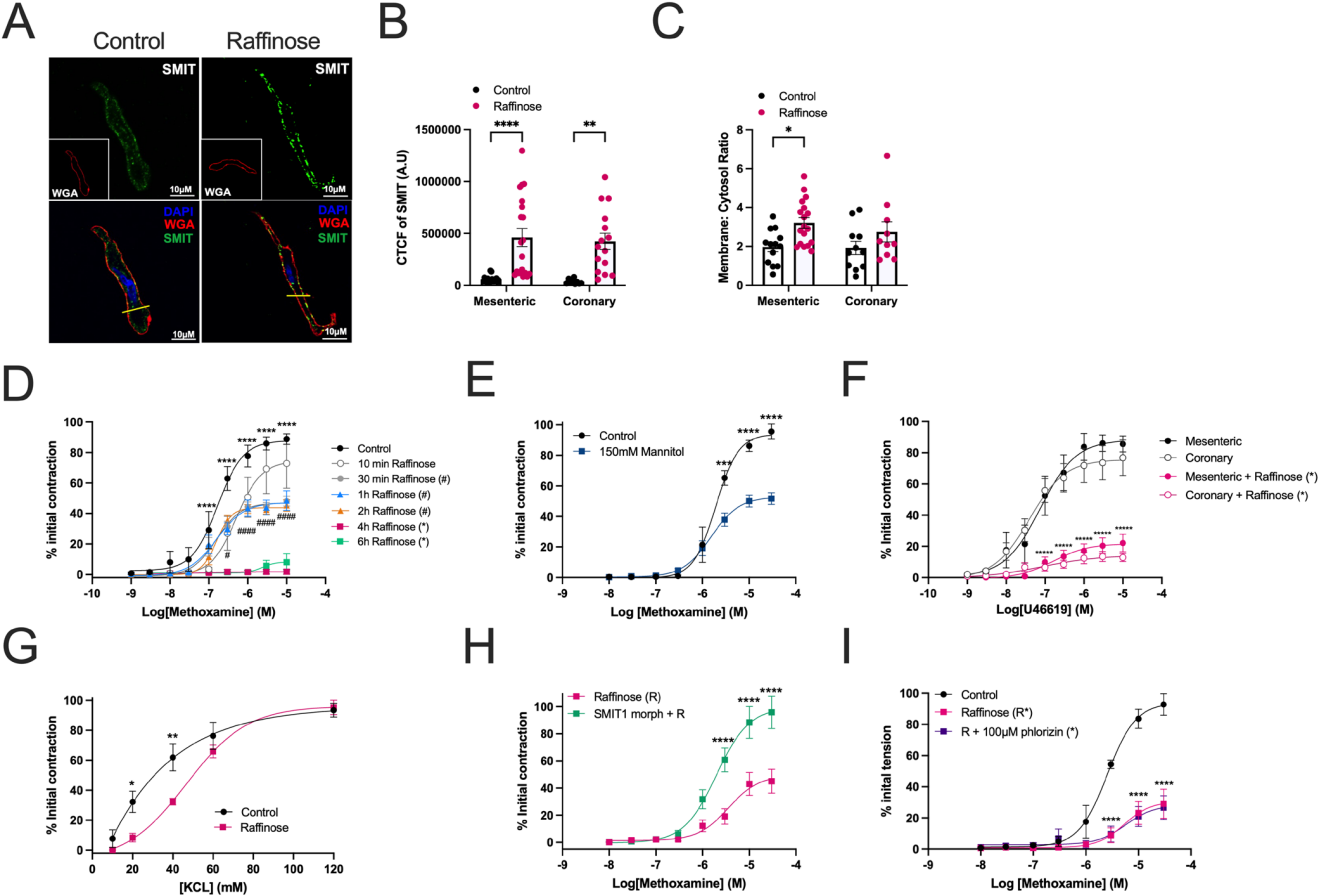
The effect of incubating in hypertonic raffinose on arterial contractile responses. A representative image of VSMCs from mesenteric arteries stained with SMIT1 (green), membrane marker, wheat germ agglutinin (WGA, red) and nucleus stain with DAPI in control (A, left) and after 4 h 150 mM raffinose (A, right). The mean corrected total cell fluorescence (CTCF) intensity of SMIT1 (B) and mean membrane cytosol ratio of SMIT1 (C) in mesenteric (*N* = 3, *n* = 15) and coronary (*N* = 3, *n* = 10) arteries incubating in control PSS (black) or 150 mM raffinose PSS (pink) for 4 h. The concentration‐dependent contraction to methoxamine in mesenteric arteries after incubating in either control PSS (black) or 150 mM raffinose for 10 min (gray), 30 min (gray filled), 1 h (blue), 2 h (orange), 4 h (pink) and 6 h (green) compared to control (black) (*N* = 5–9) shown in panel (D). (E) shows the concentration‐dependent contraction to methoxamine in mesenteric arteries in the presence (navy blue) and absence of 150 mM mannitol (*N* = 5). The mean data of the U46619‐induced contraction in control mesenteric (black) and coronary arteries (gray) and following incubation in raffinose PSS shown in panel (F) (light pink and pink *N* = 8). The mean concentration dependent response to KCL in mesenteric arteries in the presence (pink) and absence (black) of incubating with 150 mM raffinose (G). The mean concentration‐dependent contraction (mN) to methoxamine (10 nM–10 uM) in scrambled control morpholino (pink, EC_50_ = 3.56 μM) or a SMIT1 targeted morpholino (green, EC_50_ = 1.97 μM) mesenteric arteries incubated in 150 mM raffinose (4 h) is shown in panel (H) (*N* = 9). The mean contraction to methoxamine in mesenteric arteries following 150 mM raffinose incubation (pink) or 150 mM raffinose and 100 μM phlorizin (purple, I). All data points are shown as mean ± SEM denoted by error bars, and statistical values are determined by 2‐way ANOVA with multiple comparisons are calculated using post hoc Bonferroni test where *p < 0.05, **p < 0.01, ***p < 0.001, ****p < 0.0001.

The expression of SMIT1 is upregulated by a hypertonicity activated promoter element (TonEBP, [11]). Raffinose (150 mM) is a nonmetabolizable sugar that increased osmolarity from 271 to 480 mOsm. Barrese et al. (2020) [6], showed that overnight incubation of renal arteries in 150 mM raffinose increased SMIT1 fluorescence intensity in renal artery smooth muscle cells and markedly impaired methoxamine‐induced contractions compared to control PSS. We ascertained whether changes in arterial responsiveness could be elicited by shorter periods of hypertonic shocks (10 min–6 h). Incubation in raffinose containing PSS for 4 h increased both total and plasma membrane SMIT1 expression in mesenteric and coronary arteries (Figure 3A–C). Figure 3D shows that incubation in 150 mM raffinose for 10 min to 6 h followed by wash out into normal PSS inhibited the contractile response to methoxamine. This protocol is presented as a representative trace in Figure S1. Incubation in 150 mM mannitol for 1 h produced a quantitatively similar vasorelaxant effect to that caused by incubation for the same time in raffinose (Figure 3E). Incubation in 150 mM raffinose for 4 h also attenuated vasoconstrictor responses to U46619 in mesenteric and coronary arteries (Figure 3F). Raffinose treatment also reduced contractions elicited by low concentrations of KCl whereas responses to KCl concentrations above 40 mM were not affected (Figure 3G) suggesting a role for potassium channels in the anti‐contractile effect. Raffinose‐induced attenuation of methoxamine was not dependent upon the presence of a functional endothelium (Figure S2) and was prevented by knockdown of SMIT1 through translation blocking morpholinos (Figure 3H). The inhibitory effect of raffinose was not reliant upon inositol being in the bathing solution and was not affected by the SMIT1 transport inhibitor phlorizin (100 μM, Figure 3I). These data reveal a relatively rapid anti‐contractile effect of raffinose driven by increased SMIT1 expression independent of inositol transport.

**FIGURE 4:**
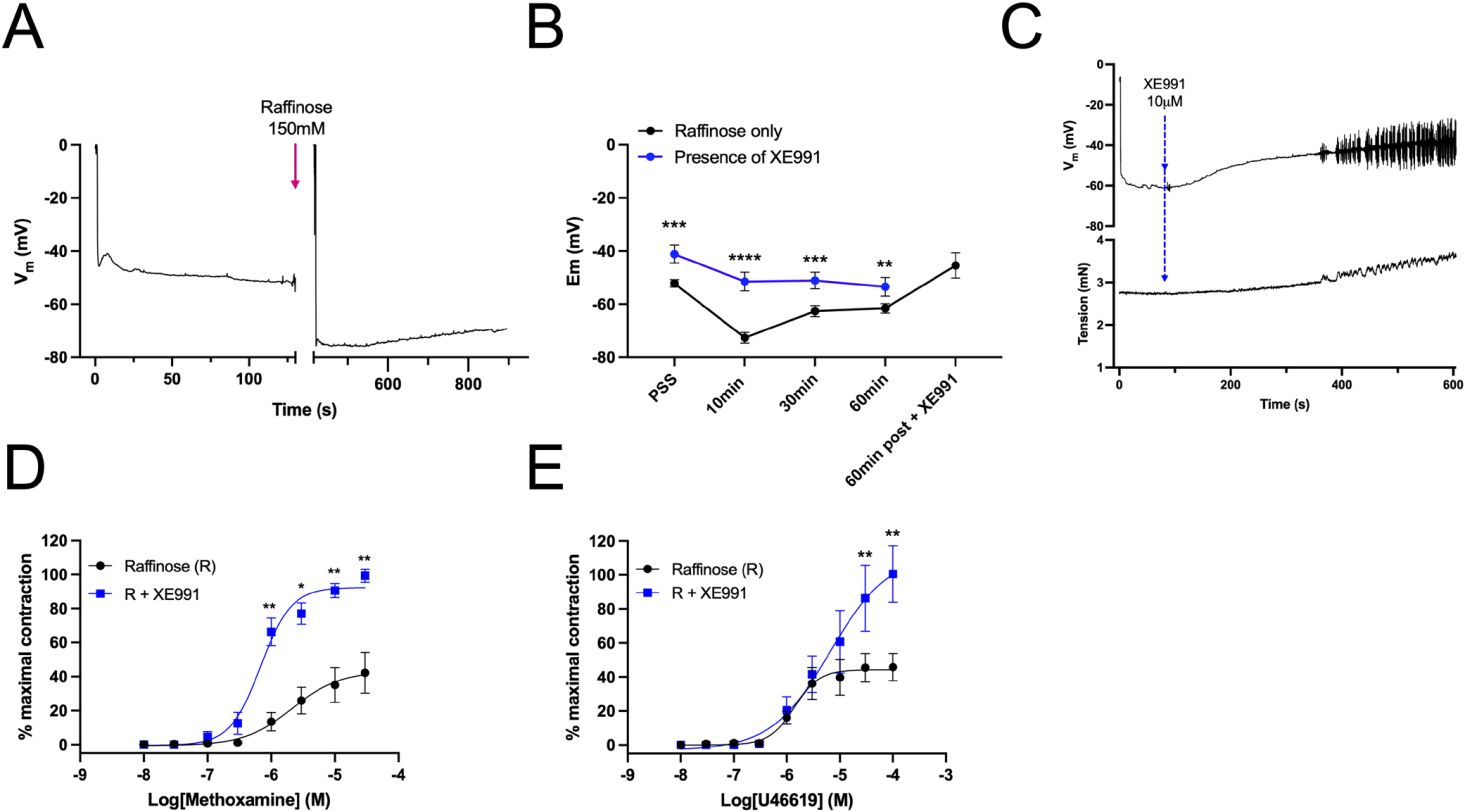
Acute raffinose incubation induces hyperpolarization in mesenteric arteries. A representative race showing simultaneous measurements of Vm before and after adding 150 mM raffinose PSS (A). The mean change in Vm before and after incubating in 150 mM raffinose for 10, 30 and 60 min in the presence (blue) and absence (black) of XE991 and application of XE991 after incubating in raffinose for 60 min (black, B). A representative trace showing the depolarization upon applying 10 μM XE991 to mesenteric arteries after incubating in 150 mM raffinose PSS for 60 min is shown in panel (C). The mean contraction to methoxamine in the presence of 150 mM raffinose (black) or raffinose in the presence of 10 μM XE991 (blue) in mesenteric (D) and coronary arteries (E) (*N* = 8). All data points are shown as mean ± SEM denoted by error bars, and statistical values are determined by 2‐way ANOVA with multiple comparisons are calculated using post hoc Bonferroni test where *p < 0.05, **p < 0.01, ***p < 0.001, ****p < 0.0001.

In a previous study, overnight incubation of renal arteries with 150 mM raffinose directly potentiated XE991‐sensitive, Kv7‐mediated K^+^ currents and produced anti‐contractile effects that were prevented by knockdown or block of Kv7 channels [6]. We speculated that a similar mechanism underlied the pro‐relaxant effects exerted by short‐term incubation in raffinose (10 min–4 h). Microelectrode recordings from mesenteric artery smooth muscle cells revealed that acute incubation with 150 mM raffinose produced membrane hyperpolarization (Figure 4A), which was prevented by the Kv7 channel blocker XE991 (Figure 4B). Similarly, incubating in a high‐sodium PSS solution with an osmolarity of 480 mOSm also produced a rapid membrane hyperpolarization (Figure S3). Application of XE991 following 60 min of raffinose incubation induced significant membrane depolarization in mesenteric vascular smooth muscle cells (Figure 4C). Moreover, XE991 prevented the raffinose‐induced attenuation of vasoconstrictor responses in mesenteric (Figure 4D) and coronary arteries (Figure 4E).

**FIGURE 5:**
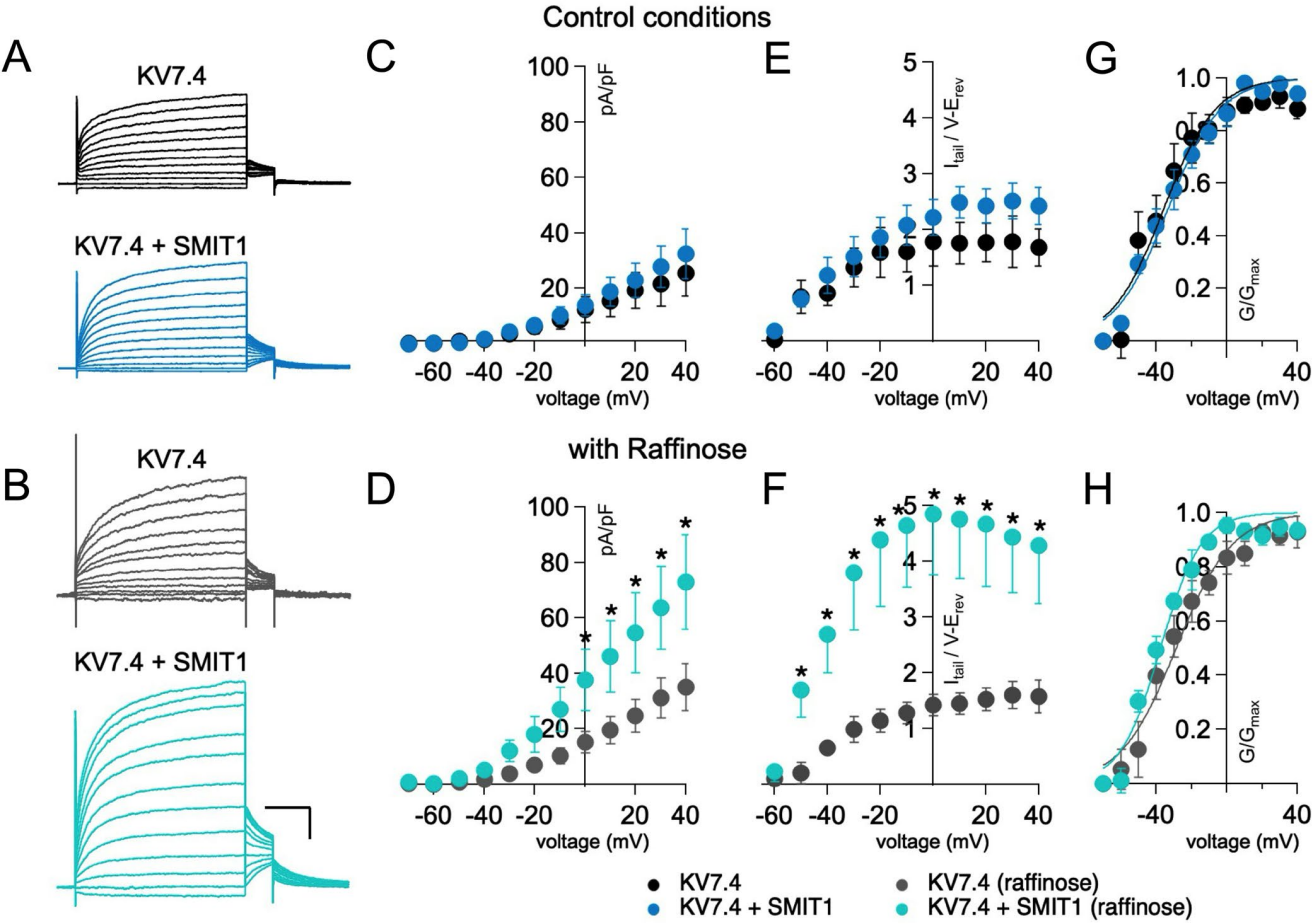
Raffionse incubation increases Kv7.4 currents in a SMIT1‐dependent manner. (A,B) Representative whole‐cell current traces of Kv7.4 obtained using a voltage‐step protocol (960 ms) spanning −70 mV to +40 mV in 10 mV increments, after which the voltage was returned to −40 mV, from a holding potential of −60 mV. Kv7.4 alone (black) and Kv7.4 co‐expressed with SMIT1 (blue) under control conditions, as well as Kv7.4 (gray) and Kv7.4 + SMIT1 (light blue) under hyperosmotic conditions after 1 h incubation in DMEM supplemented with 150 mM raffinose. (C,D) Current density–voltage relationship of Kv7.4 and Kv7.4 + SMIT1 under control conditions (black and blue) and after 1 h treatment with raffinose (gray and light blue). Two‐way ANOVA with Bonferroni post hoc test (*p < 0.05). (E,F) Absolute conductance–voltage relationship of Kv7.4 and Kv7.4 + SMIT1 under control conditions (black and blue) and under hypoosmotic conditions after 1 h treatment with raffinose (gray and light blue). Two‐way ANOVA with Bonferroni post hoc test (*p < 0.05). (G,H) Mean normalized conductance (*G*/*G*_max_) plotted against voltage for each condition and fitted with a Boltzmann function. Kv7.4 (control, black) *V*½ = −35.8 ± 2.6 mV; Kv7.4 + SMIT1 (control, blue) *V*½ = −33.4 ± 1.5 mV; Kv7.4 (raffinose, gray) *V*½ = −28.7 ± 2 mV; KV7.4 + SMIT1 (raffinose, light blue) *V*½ = −37.5 ± 1.6 mV. Two‐way ANOVA with Bonferroni post hoc test showed no significant differences.

A series of experiments on heterologously expressed Kv7.4 were perfomed to determine if hypertonicity increased Kv7 channels directly or indeed relied upon SMIT1. Under control conditions, both current density and conductance showed a slight increase in cells co‐expressing Kv7.4 and SMIT1 compared to Kv7.4 alone; nevertheless, these differences were not statistically significant. Currents generated by Kv7.4 expression alone were not altered by incubation in DMEM supplemented with 150 mM raffinose for 1 h (Figure 5A–C). However, currents generated by the expression of Kv7.4 + SMIT1 exhibited a marked increase in current density (*p* < 0.001, Figure 5E) and conductance values (*p* < 0.001, Figure 5F) after 1 h incubation in raffinose. Post hoc analysis (Bonferroni correction) revealed that SMIT1 significantly increased current density at voltages ≥ 0 mV, while significant differences in conductance were observed from −40 mV onwards (*p* < 0.001). There was also a leftward shift in the activation curve with V½ changing from −28.7 ± 2 mV to −37.5 ± 1.6 mV (*p* = 0.13), indicating a trend toward activation at more hyperpolarized potentials. Thus, augmentation of Kv7.4 by external hypertonicity was transduced by SMIT1.

**FIGURE 6:**
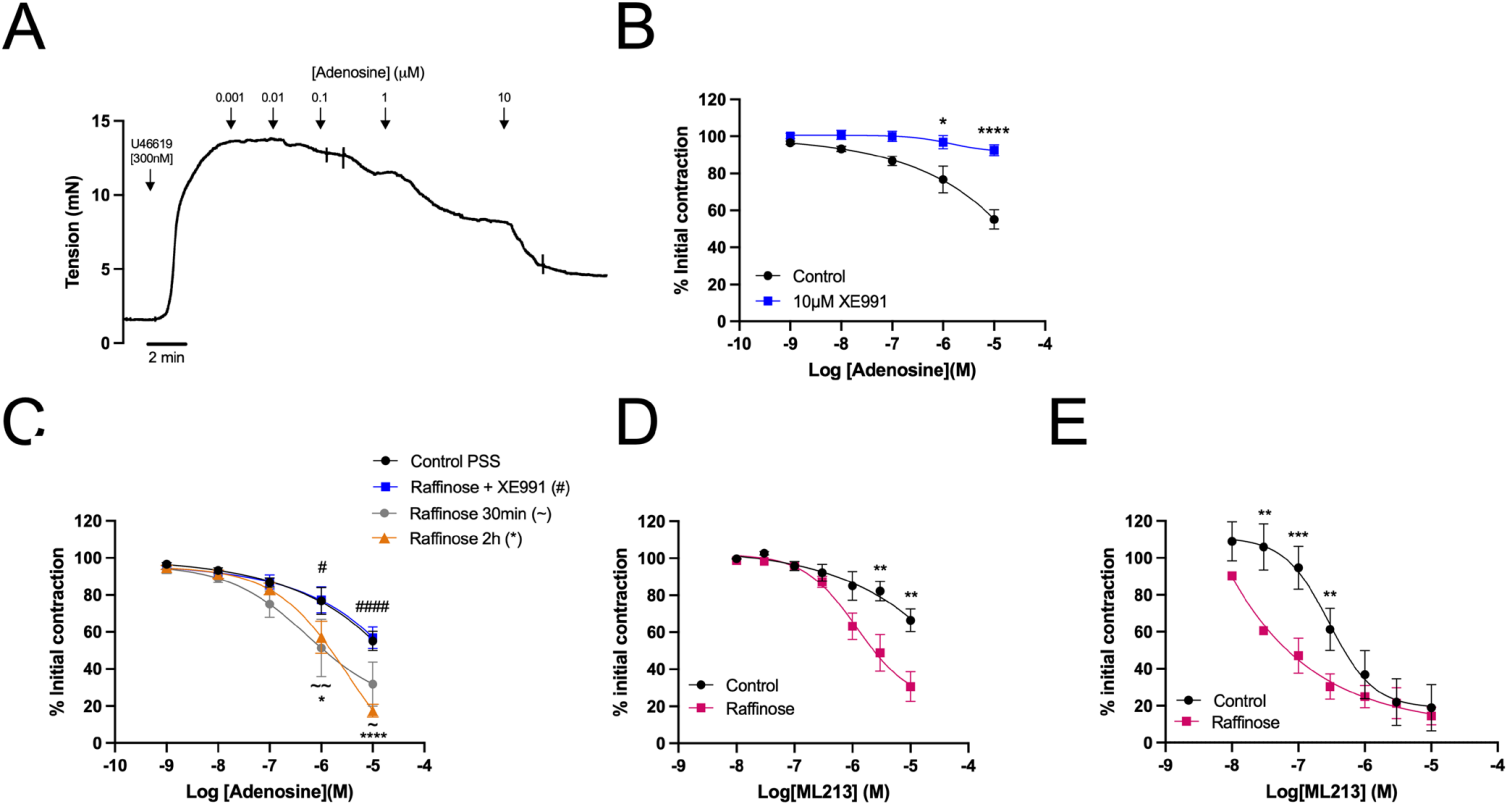
Adenosine and ML213‐induced relaxations are enhanced in mesenteric arteries following incubation in 150 mM raffinose. A representative trace showing the relaxation to Adenosine (1 nM–10 μM) in control PSS (A). The mean data of adenosine‐induced relaxation in the presence (blue) and absence (black) of 10 μM XE991 (B). The mean concentration‐dependent relaxation to adenosine (1 nM–10 μM) in mesenteric arteries when incubated in either control PSS (black) or 150 mM raffinose PSS for 30 min (grey), 2 h (orange) or 150 mM raffinose and 10 μM XE991 (blue) for 2 h shown in panel (C). The mean response to ML213 in mesenteric arteries (D) and coronary arteries (E) pre‐contracted with 300 nM U46619 in the presence (pink, *N* = 6) and absence (black, *N* = 6) of 150 mM raffinose. All data points are shown as mean ± SEM denoted by error bars with *N* = 5, and statistical values are determined by 2‐way ANOVA with multiple comparisons are calculated using post hoc Bonferroni test where *p < 0.05, **p < 0.01, ***p < 0.001, ****p < 0.0001.

As SMIT1 knockdown impaired responses to Gs‐linked receptors we speculated that boosting SMIT1 membrane expression would augment receptor‐mediated vasodilation. Adenosine relaxes arteries through Gs‐coupled A2a/b receptors [22] and is far less effective in mesenteric arteries compared to coronary arteries. In control mesenteric arteries adenosine produced a concentration‐dependent relaxation that reached approximately 50% at 10 μM, which was prevented by prior application of 10 μM XE991 (Figure 6A,B). Incubation with raffinose for 30 min and 2 h enhanced adenosine‐induced relaxations that were prevented by XE991 (Figure 6C). The relaxant response to ML213 [23, 24, 25] in mesenteric and coronary arteries was also augmented by short‐term (1 h) incubation in raffinose (150 mM; Figure 6D,E).

Thus, hypertonicity produced a graded and rapidly occurring pro‐relaxant effect associated with increased Kv7 channel‐dependent membrane hyperpolarization that was reliant upon SMIT1.

### 3.3 SMIT1 Associates With Kv7.4/5 Channels and Gβγ Subunit to Evoke Relaxation

**FIGURE 7:**
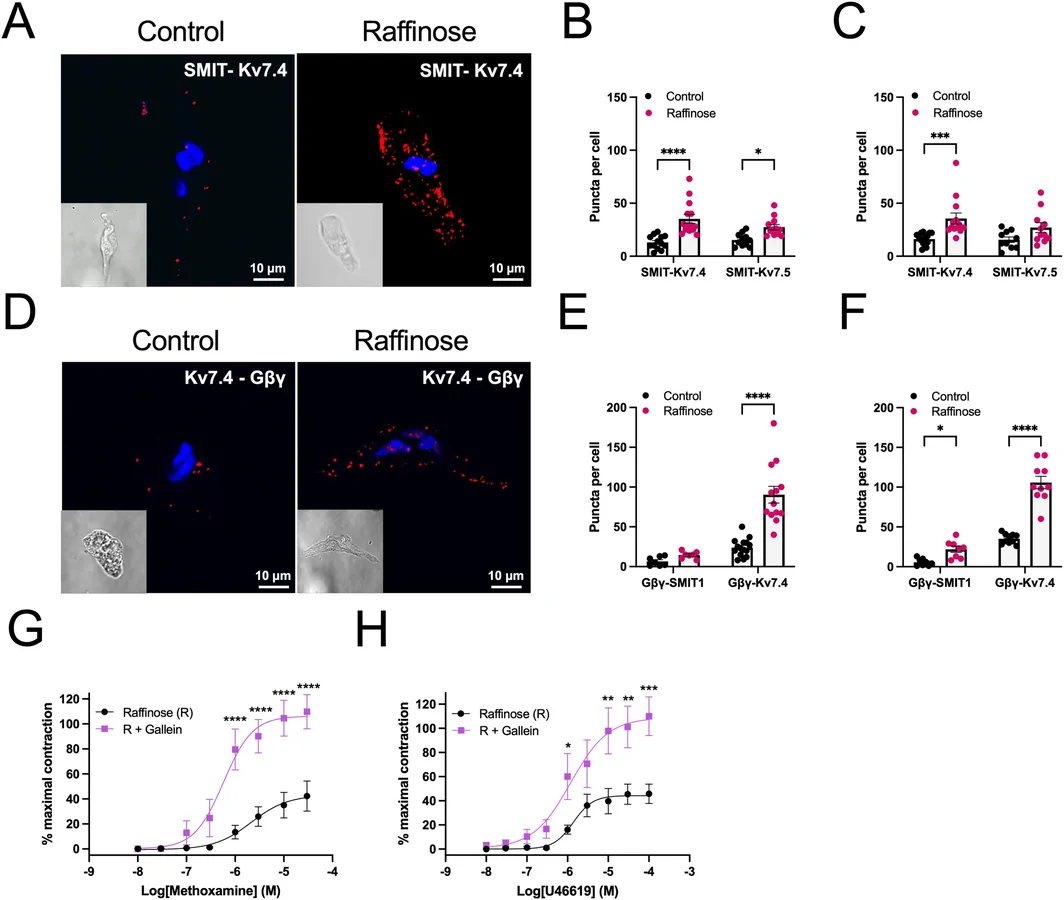
The co‐localisation and role of Kv7 channels and Gβγ in SMIT1 induced relaxations. PLA puncta (red) show the co‐localisation of SMIT with Kv7.4 in mesenteric VSMCs incubated in either control PSS or 150 mM raffinose PSS for 4 h (A). Bar graph showing the mean number of PLA puncta for SMIT‐Kv7.4 and SMIT‐Kv7.5 in mesenteric (B) (*N* = 3 *n* = 15) and coronary (C) (*N* = 3 *n* = 15) VSMCs incubated in either control PSS (black) or 150 mM raffinose PSS (pink) for 4 h. PLA puncta (red) show the co‐localisation of Kv7.4 with Gβγ in mesenteric VSMCs incubated in either control PSS or 150 mM raffinose PSS for 4 h (D). Bar chart showing the mean number of Gβγ‐Kv7.4 and Gβγ‐SMIT1 PLA puncta per cell in mesenteric (E, *N* = 3 *n* = 14) and coronary (F, *N* = 3 *n* = 10) arteries incubated in either control PSS (black) or 150 mM raffinose PSS (pink). The mean contraction to methoxamine in the presence of 150 mM raffinose (black) or raffinose in the presence of 50 μM Gallein (purple) in mesenteric (G) and coronary arteries (H) (*N* = 8). All data points are shown as mean ± SEM denoted by error bars, and statistical values are determined by 2‐way ANOVA with multiple comparisons are calculated using post hoc Bonferroni test where *p < 0.05, **p < 0.01, ***p < 0.001, ****p < 0.0001. (*N* = number of animals used, *n* = number of technical repeats).

Having established that hypertonic extracellular solution produced a rapid anti‐contractile effect, experiments were performed to investigate the underlying mechanisms. Proximity ligation assay established that in mesenteric artery smooth muscle cells, SMIT1 conjoined with Kv7.4 and Kv7.5 and this association was augmented following short‐term incubation in 150 mM raffinose (Figure 7A,B). Similar data was derived from coronary artery smooth muscle cells (Figure 7C).

Gβγ subunits have previously been shown to associate with Kv7.4 and enhance channel activity [26, 27], this interaction is crucial for the manifestation of several receptor‐mediated relaxations [28]. PLA revealed that Gβγ was closely associated with both Kv7.4 and SMIT1 at high levels in mesenteric and coronary arterial smooth muscle cells and raffinose incubation increased this association in both artery types (Figure 7D–F). Thus, extracellular hypertonicity enhanced the association of Kv7 channels with the positive regulatory proteins SMIT1 and Gβγ. In support of this, the Gβγ inhibitor gallein (50 μM) prevented raffinose‐induced inhibition of U46619 and methoxamine‐induced contractions (Figure 7G,H).

### 3.4 SMIT1 Pro‐Relaxant Effects Are Mediated by Serum Glucocorticoid Kinase 1 (SGK1)

Incubation with raffinose impaired receptor‐mediated contraction within 10 min that was not consistent with a hypertonicity‐mediated change in SMIT1 gene transcription and subsequent increased protein production. Klaus et al. (2008) [10] showed that SMIT1 membrane abundance was limited by Nedd4‐2‐mediated ubiquitination, which was downregulated following phosphorylation by serum/glucocorticoid‐dependent kinase 1 (SGK1).

**FIGURE 8:**
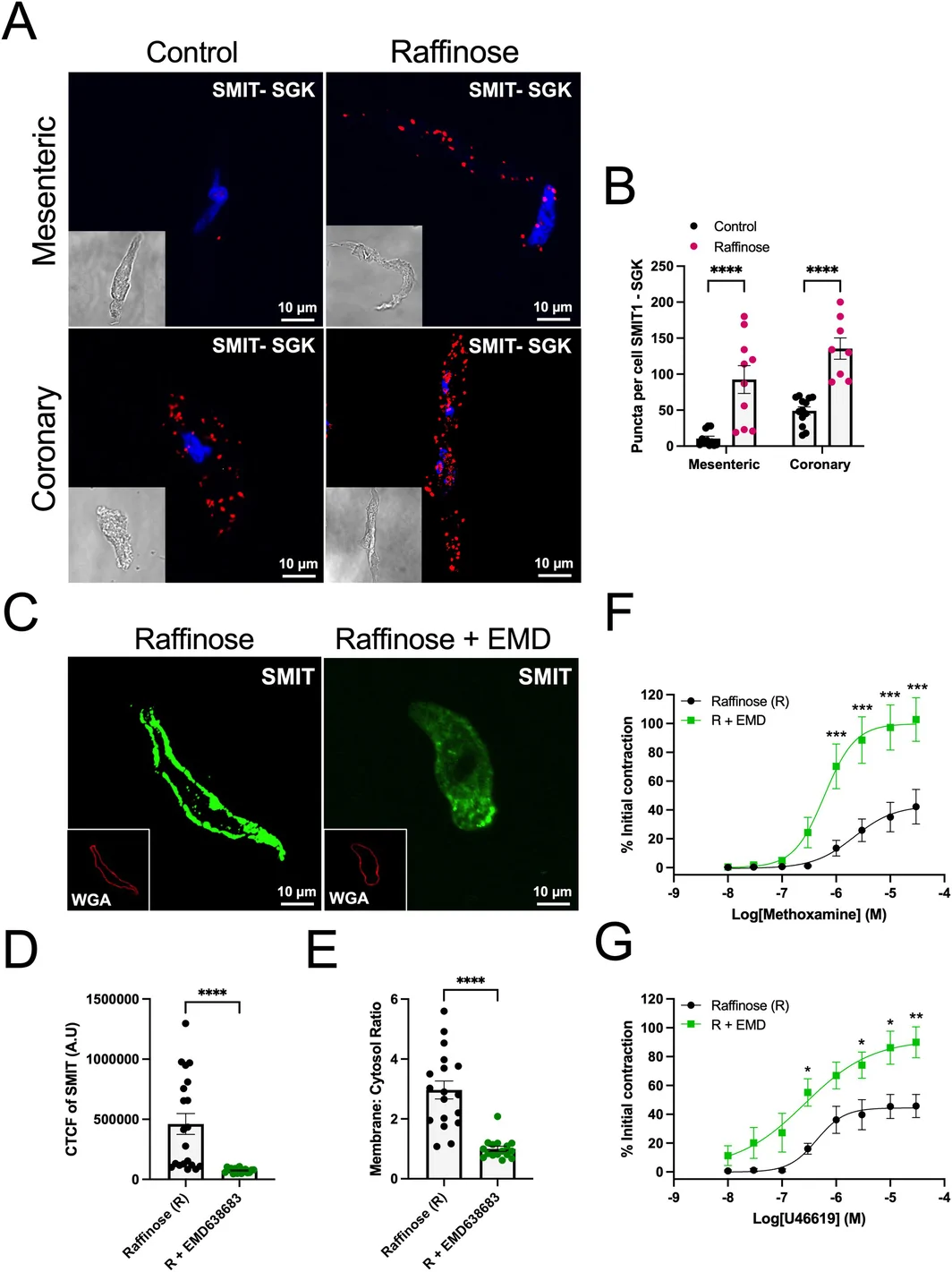
The role of SGK in regulating SMIT1 membrane expression and activity. SMIT‐SGK PLA puncta staining (red) in mesenteric arteries and coronary arteries incubated in either control PSS or 150 mM raffinose PSS (A). The mean number of PLA puncta in mesenteric (*N* = 3 *n* = 10) and coronary (*N* = 3 *n* = 10) arteries following 4‐h control PSS (black) or 150 mM raffinose (pink) incubation in male rats is shown in panel (B). Representative staining of SMIT1 (green) in raffinose (C, left) and in raffinose with SGK inhibitor EMD638683 (C, right) with the membrane marker, wheat germ agglutinin (WGA, red) and nucleus stain with DAPI (blue). The mean total cell fluorescence intensity (CTCF) of SMIT1 in mesenteric VSMCs incubated in raffinose alone (black, *N* = 4 *n* = 20) or with an SGK1 inhibitor EMD638683 (green, *N* = 4 *n* = 20) is shown in panel (D). The mean membrane: Cytosol ratio of SMIT1 staining in mesenteric VSMCs incubated with raffinose alone (black, *N* = 4 *n* = 18) or with EMD638683 (green, *N* = 3 *n* = 16) is shown in panel (E). The contraction to methoxamine (10 nM–10 μM) following 4‐h incubation in 150 mM raffinose in the presence (green) and absence (black) of SGK inhibitor EMD638683 in mesenteric (F, *N* = 7) and coronary (G, *N* = 7) arteries. All data points are shown as mean ± SEM denoted by error bars, and statistical values are determined by 2‐way ANOVA with multiple comparisons are calculated using post hoc Bonferroni test, and for (D,E) statistical values are determined by a Mann–Whitney test where *p < 0.05, **p < 0.01, ***p < 0.001, ****p < 0.0001. (*N* = number of animals used, *n* = number of technical repeats).

We therefore investigated whether SGK1 contributed to the rapid SMIT1‐dependent vasorelaxant effects upon hypertonicity. Proximity ligation assays showed that SGK1 associated with SMIT1, which increased following incubation in 150 mM raffinose in both mesenteric and coronary vascular smooth muscle cells (Figure 8A,B). Pre‐incubation with the SGK1 inhibitor EMD638683 prevented the raffinose‐induced increase in SMIT1 membrane abundance (Figure 8C–E). EMD638683 also attenuated the raffinose‐induced inhibition of methoxamine in mesenteric (Figure 8F) and coronary arteries (Figure 8G). Thus, rapid alterations in arterial responsiveness produced by hypertonic media appear to be mediated by SGK1 recruitment limiting SMIT1 removal from the plasma membrane.

### 3.5 SMIT1 Modulates Arterial Contractility in Female Arteries

**FIGURE 9:**
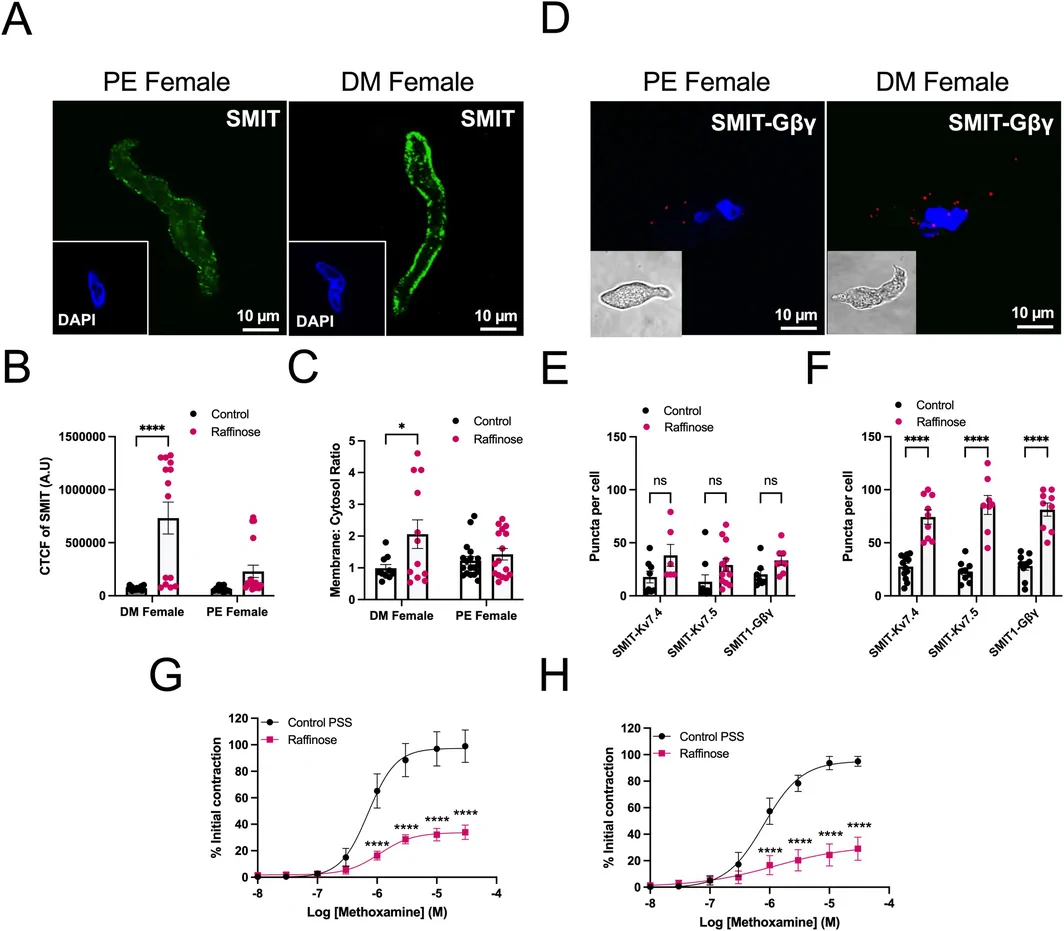
The expression and function of SMIT1 in mesenteric arteries from female rats. A representative image of mesenteric smooth muscle cells stained with SMIT1 (green) and nucleus stain with DAPI in PE female (A, left) and DM female rats (A, left) with and nucleus stain with DAPI (blue). The mean corrected total cell fluorescence (CTCF) intensity of SMIT1 staining in mesenteric arteries from DM female and PE female rats with (pink) and without (black) incubating in 150 mM Raffinose for 4 h (B). The mean membrane cytosol ratio of SMIT1 in mesenteric arteries from DM female and PE female rats in control vessels (black) or vessels incubated with 150 mM raffinose for 4 h (pink, C). Proximity ligation assay staining of SMIT‐Gβγ puncta in vascular smooth muscle cells from PE and DM female mesenteric arteries with and nucleus stain with DAPI (blue, D). Bar charts showing PLA puncta of SMIT1‐Kv7.4, SMIT‐Kv7.5 and SMIT‐Gβγ co‐localisations in mesenteric arteries from PE female (E, *N* = 2 *n* = 8) and DM female (F, *N* = 3 *n* = 9) rats following incubation for 4 h in control PSS (black) or 150 mM raffinose PSS (pink). The mean concentration‐dependent contraction to methoxamine in mesenteric arteries from PE (G) and DM female (H) rats incubated in either control PSS (black) or 150 mM raffinose PSS (pink) (*N* = 9). All data points are shown as mean ± SEM denoted by error bars and statistical values are determined by 2‐way ANOVA with multiple comparisons are calculated using post hoc Bonferroni test where *p < 0.05, **p < 0.01, ***p < 0.001, ****p < 0.0001.

There is considerable evidence that arterial regulation is sex‐dependent. Recently, Baldwin et al. (2023) [17, 21] reported that Kv7.2–7.5 activators relaxed several arteries that were influenced by the estrous cycle stage. We assessed the impact of raffinose in mesenteric and coronary arteries from female rats at different stages of the estrous cycle, segregating them into the higher‐estrogen proestrus/estrus (PE) or lower‐estrogen diestrus/metestrus (DM) according to [21]. Total SMIT1 abundance was significantly higher in mesenteric arteries from DM females compared to PE females and male rats (Figure 9A–C). In addition, PLA punctae for SMIT1 with Kv7.4, Kv7.5 or Gβγ were higher in smooth muscle cells from DM rats compared to PE females (Figure 9D) and raffinose enhanced the number of associations in cells from DM but not PE rats (Figure 9E,F). A similar pattern of SMIT1 association with Kv7 channels and Gβγ was observed in coronary artery smooth muscle cells (Figures S4 and S5). Overall, SMIT1 abundance and association with Kv7 channels and Gβγ exhibit estrous cycle dependence.

## 4 Discussion

The interaction between SMIT1 and Kv7 ion channel proteins has been documented in the choroid plexus epithelium, neurons, overexpression systems and more recently arteries demonstrating reciprocal regulation of each other's activity independent of myo‐inositol transport [2, 3, 4, 5, 6, 7]. The present study reveals the sodium/myo‐inositol transporter SMIT1 to be a crucial regulator of arterial reactivity independent of inositol transport, which provides a rapid mechanism for arterial smooth muscle to respond to external stimuli. This is based upon an accumulation of different strands of data. We show that incubation of arteries in moderately hypertonic solution (150 mM raffinose and mannitol, 480 mOSm) led to rapid inhibition of receptor‐mediated contractions but not high‐potassium‐induced responses in mesenteric arteries. The anti‐contractile effect was associated with marked hyperpolarization of the smooth muscle membrane potential and prevented by molecular knockdown of SMIT1. Conversely, adenosine produced robust relaxations of mesenteric arteries after short‐term incubation with raffinose that were not present in control arteries and the Kv7 activator ML213 was considerably more potent. Short‐term incubation of coronary arteries in raffinose also led to impaired contractile responses to U46619 and methoxamine but augmented relaxations to ML213. The membrane potential hyperpolarization and pro‐relaxant response of raffinose was counteracted by the Kv7 blocker XE991. Patch clamp experiments on heterologously expressed Kv7.4 channels revealed that 1 h incubation in raffinose only augmented currents if SMIT1 was co‐expressed. Proximity ligation assays in arterial smooth muscle revealed that raffinose increased association of SGK1 with SMIT and Gβγ with Kv7 channels. The Gβγ blocker gallein and the SGK1 inhibitor, EMD638683 also suppressed the anti‐contractile effect of raffinose. These cumulative data reveal that short‐term increase in extracellular tonicity translates to an anti‐contractile phenotype in arteries mediated by SGK1 preventing SMIT1 removal from the cell membrane and interacting with Kv7 channels. The subsequent hyperpolarization limits the open probability of *L*‐type and *T*‐type voltage‐dependent calcium channels in the arterial smooth muscle contributing to reduced contraction or relaxation.

### 4.1 SMIT1 Regulates Arterial Contractility

Overnight incubation (~16 h) of renal arteries with hypertonic 150 mM raffinose increased the membrane abundance of SMIT1 and reduced the contractile response to methoxamine and low concentrations of KCl (< 40 mM) [6]. The present study shows that reduction of receptor‐mediated vasocontraction and increase in SMIT1 expression did not require prolonged incubation in raffinose with significant effects observed after 30 min indicating that SMIT1 can rapidly modulate arterial tone. These findings align with earlier work indicating that acute exposure to hypertonic solutions whether evoked by sucrose, mannitol, or high glucose‐induced vasorelaxation in several arterial beds including skeletal muscle arterioles, cerebral and coronary arteries through K^+^ channel activation [29, 30, 31, 32, 33, 34, 35].

Acute incubation with raffinose hyperpolarized the resting membrane potential of mesenteric vascular smooth muscle cells, an effect prevented by the Kv7‐channel blocker XE991. Single‐cell electrophysiological experiments showed that raffinose only augmented Kv7.4 channel currents if SMIT1 was co‐expressed, confirming that rapid Kv7 activation under hypertonic conditions was due to increased SMIT1 expression. Consistent with this, short‐term (1 h) exposure to raffinose and 2 h exposure to mannitol significantly enhanced relaxations to adenosine and to the Kv7 activator ML213 in both mesenteric and coronary arteries. Conversely, morpholino‐based knockdown of SMIT1 protein markedly impaired relaxations to ML213, as well as isoprenaline and CGRP, which are mediated partially by recruitment of Kv7 channels [28, 36]. Together these findings indicate SMIT1 is required for full Kv7.4/7.5 channel function and acts as a positive regulator of Kv7‐mediated vasodilation, which is consistent with previous studies showing SMIT1 interacts with Kv7.2/3 and Kv7.1 ion channels in neurons and the choroid plexus [3, 4, 5, 7] and the presence of SMIT1 negatively shifts the voltage dependence of these ion channels' currents and enhances rate of activation [5].

Myo‐inositol, transported by SMIT1, is a precursor for phosphatidylinositol 4, 5‐bisphosphate (PIP_2_), a key lipid cofactor required for stabilizing Kv7 channel opening and promoting activation. Altering SMIT activity has been shown to regulate neuronal excitability by modulating PIP_2_ levels [3]. Therefore, the inhibitory effects of raffinose in the present study may reflect increased SMIT1‐dependent myo‐inositol transport and enhanced PIP_2_‐dependent activation of Kv7.4/7.5 channels. However, no inositol was present in the external solution during the acute experiment or incubation periods. Moreover, the non‐selective inhibitor of SMIT, phlorizin, does not prevent the anti‐ contractile effects produced by raffinose in renal artery [6] and present study. Thus, the effects of SMIT1 on Kv7 channels were not due to myo‐inositol uptake increasing intracellular PIP_2_ and instead involved direct modulation of the Kv7 channel protein, as indicated by [5]. Our over‐expression studies show that channel conductance increased with a small negative shift in voltage‐dependence that aligns with the observation for SMIT1 physical interaction with the pore module of Kv7.1 and Kv7.2 [5].

### 4.2 SMIT1 Resides Close to Kv7.4/5 and Gβγ Proteins Contributing to Vasorelaxation

Co‐immunoprecipitation assays have shown that SMIT1 interacts with Kv7.2 by binding to the S5‐6 subunits of the pore‐forming region, altering ion selectivity and channel sensitivity [5]. In the present study, SMIT1 was found in close association with Kv7.4 and Kv7.5 and this association was augmented by short‐term incubation in 150 mM raffinose in both mesenteric and coronary arteries. In patch‐clamp studies on heterologously expressed Kv7.4 channels, the effect of raffinose was reliant upon SMIT1 expression, did not require inositol to be present and was manifest as an increase in current density with minimal change in voltage dependence of activation consistent with a physical interaction leading to altered conduction. Gβγ subunits have been identified as crucial regulators of the Kv7.4 ion channel, with Gβγ and Kv7.4 associated in close proximity in arterial smooth muscle, internal Gβγ enrichment enhancing heterologously expressed Kv7.4 and native Kv7 current, and various Gβγ blockers including gallein abolishing the voltage‐dependent activity of Kv7.4 channels [26]. The present study showed that Gβγ associated with Kv7.4/7.5 and SMIT1 in coronary and mesenteric artery smooth muscle cells from male and female rats, and these associations increased following a 4‐h incubation in raffinose‐enriched PSS. Moreover, gallein prevented the raffinose‐induced reduction in U46619‐evoked contractions in mesenteric arteries to a similar extent as the Kv7 inhibitor XE991. As SMIT1 associates with Gβγ, we propose that the transporter coordinates the G‐proteins to alter Kv7.4/7.5 activity. Hence, Kv7 ion channel proteins, SMIT and Gβγ form a complex to regulate arterial tone. As Kv7 channels in smooth muscle cells are modulated by the single transmembrane auxiliary subunit KCNE 4 [18] and KCNE proteins modify the effect of SMIT1 on Kv7 channels [4, 5] there is an extra layer of complexity to consider in this signaling complex. Future studies will focus on deciphering the complex signal interactions of SMIT‐Kv7‐KCNE4 and Gβγ subunits.

### 4.3 SGK1 Regulates SMIT1 Expression Modulating Its Effect on Arterial Contractility

The anticontractile effect of increasing SMIT1 may be due to the raffinose‐mediated activation of the transcription factor TonEBP (tonicity‐responsive enhancer‐binding protein), resulting in increased SMIT1 gene expression [11, 37]. However, activation of TonEBP in response to hypertonicity is slow, with a detectable increase only occurring after approximately 3 h [11]. The present study observed that the effect of raffinose on contractility manifested rapidly, with U46619‐ and methoxamine‐induced contractions being impaired even after 10 min of incubating in high raffinose PSS. Therefore, other regulators of SMIT1 are contributing to its increased membrane protein abundance, resulting in the observed anticontractile effects.

Serum‐and glucocorticoid‐inducible kinases (SGK1‐3) are upregulated by hypertonicity [38, 39] and are a known post‐translational regulator of numerous ion channels [40, 41, 42, 43, 44, 45] either directly or through regulation of membrane abundance. For instance, SGK modulates the expression of hERG [43, 44] and NaV1.5 [40], proteins in myocardial cell membranes by deactivating the ubiquitin ligase Nedd4‐2, thereby preventing the retrograde trafficking of these channels and increasing their membrane abundance. Electrophysiological studies in Xenopus oocytes identified that SMIT1‐mediated myo‐inositol transport increased when SGK1 was co‐expressed due to increased residency of SMIT1 in the membrane [10], following phosphorylation and deactivation of Nedd4‐2, which removed SMIT1 from the cell membrane. SGK can also phosphorylate SMIT1 per se, leading to further SMIT1 membrane expression [10]. The present study shows that the anti‐contractile effect in raffinose incubated arteries was prevented by XE991, gallein and the SGK1 inhibitor, EMD638683. Strikingly, SGK1 was also found to be in close proximity with SMIT in mesenteric and coronary arteries, with the number of associations increasing upon raffinose incubation. Our data suggest that the rapid impairment of arterial contractility and pro‐relaxant arterial state produced by hypertonicity was caused by upregulated SGK activity enhancing SMIT1 protein expression [10] and subsequently increasing SMIT1 associations with Kv7.4/5 channels. Nedd4‐2 also influences Kv7.1 removal from the cell membrane so SGK1 may also prevent Kv7 channel degradation and SGK1 also phosphorylates Kv7 channels directly [46, 47, 48, 49], which could also increase the membrane abundance of Kv7.4/7.5 in the vascular smooth muscle. Our electrophysiological data suggest the presence of SMIT1 is crucial for linking extracellular change into cellular effect.

### 4.4 Oestrus Cycle Differences in SMIT1‐Induced Relaxations

The present study demonstrated that the expression of SMIT1 in the presence and absence of 150 mM raffinose was considerably lower in smooth muscle cells from mesenteric and coronary arteries from PE females compared to male and DM females, with DM females consistently having the greatest total fluorescence intensity of SMIT1 protein. Furthermore, DM females consistently exhibited a greater number of SMIT1 associations with Kv7.4, Kv7.5 and Gβγ compared to males and PE females, with PE females displaying considerably fewer SMIT1 co‐localization events both in the presence and absence of raffinose. Collectively, these findings suggest that estrous cycle‐dependent differences exist in the expression and molecular associations of SMIT1 within DM female and PE female arterial tissue. Baldwin et al. [17, 21], identified that Kv7 activators were less effective relaxants in arteries from PE females compared to DM females due to a reduction in the forward trafficking of Kv7.4 via HSP90 leading to a reduction in Kv7.4 expression in the VSMC membrane [17, 21]. The present study demonstrates that sex‐ and estrous cycle‐dependent differences in Kv7 responsiveness may arise as a consequence of altered protein–protein associations within vascular smooth muscle cells.

## 5 Conclusion

This study supports the concept that SMIT1 is a key component of Kv7.4/7.5 ion channel complexes in vascular smooth muscle, where it regulates arterial contractility and vasodilation in mesenteric and coronary arteries from both male and female rats. SMIT1 facilitates the association of Gβγ subunits with Kv7.4/7.5 channels and, through a rapid SGK1‐dependent mechanism, transduces environmental stimuli into changes in arterial responsiveness. Collectively, these findings define a novel paradigm in arterial regulation centered on the SMIT1–Kv7 chansporter complex, broadening our fundamental understanding of the molecular mechanisms that govern vascular smooth muscle function and arterial tone.

## Author Contributions

Elizabeth A. Forrester generated and analyzed data, and wrote the manuscript. Vincenzo Barrese and Anthony P. Albert reviewed the manuscript and contributed to experimental design. Miguel Benitez‐Angeles did the patch clamp studies. Skye Lau did new functional experiments. Christopher J. Garland did the microelectrode recordings. Iain A. Greenwood supervised the project, edited the manuscript, and provided the funding for the project.

## Funding

This work was supported by a PhD studentship for Elizabeth A. Forrester. (FS/PhD/21/2912) from the British Heart Foundation awarded to Iain A. Greenwood.

## Conflicts of Interest

The authors declare no conflicts of interest.

## Supporting information


**Figure S1:** Raffinose 4 h incubation protocol: A representative trace of the response to methoxamine (10 nM–30 μM) in mesenteric arteries before and after incubating for 4 h in control PSS (black) and 150 mM raffinose (pink).

**Figure S2:** The concentration‐dependent contraction to U46619 after 4 h of raffinose incubation in mesenteric arteries with either positive endothelium or denuded.
The mean response to U46619 when incubated in 150 mM raffinose in mesenteric arteries with either positive endothelium (EC+, pink) or denuded (EC−, Blue) compared to control PSS incubated vessels (black). All data points are shown as mean + ‐SEM denoted by error bars with *N* = 5, and statistical values are determined by two‐way ANOVA with multiple comparisons are calculated using post hoc Bonferroni test where *p < 0.05, **p < 0.01, ***p < 0.001, ****p < 0.0001.

**Figure S3:** Effect of 480 mOSm high Na Krebs on smooth muscle membrane potentential.
The mean change in Vm in control PSS and after incubating in 480 mOSm high Na Krebs for 30 min (green). All data points are shown as mean + ‐SEM denoted by error bars with *N* = 5, and statistical values are determined by paired T test where *p < 0.05.

**Figure S4:** The expression and function of SMIT1 in coronary arteries from female rats.
The mean corrected total cell fluorescence (CTCF) intensity of SMIT1 staining in coronary arteries from DM female and PE female rats with (pink) and without (black) incubating in 150 mM Raffinose for 4 h (A). The mean membrane cytosol ratio of SMIT1 in coronary arteries from DM female and PE female rats in control vessels (black) or vessels incubated with 150 mM raffinose (pink) for 4 h (B). Bar charts showing PLA puncta of SMIT1‐Kv7.4, SMIT‐Kv7.5 and SMIT‐Gβγ co‐localization in coronary arteries from DM female (C, *N* = 3 *n* = 9) and PE female (D, *N* = 2 *n* = 8) rats following incubation for 4 h in control PSS (black) or 150 mM raffinose PSS (pink). All data points are shown as mean + ‐SEM denoted by error bars and statistical values are determined by 2‐way ANOVA with multiple comparisons are calculated using post hoc Bonferroni test where *p < 0.05, **p < 0.01, ***p < 0.001, ****p < 0.0001.

**Figure S5:** Expression and co‐localisation of SMIT1 with Kv7.4, Kv7.5 and Gβγ in male and female mesenteric and coronary arteries.
The mean corrected total cell fluorescence (CTCF) intensity of SMIT1 staining in mesenteric (A) and coronary (C) arteries from male (black), DM female (blue) and PE female (red) rats with and without incubating in 150 mM Raffinose for 4 h. The mean membrane cytosol ratio of SMIT1 in mesenteric (B) and coronary (D) arteries from male (black), DM female (blue) and PE female (red) rats in control vessels or vessels incubated with 150 mM raffinose for 4 h.
Proximity ligation assays (PLA) showing the mean number of SMIT‐Kv7.4, SMIT‐Kv7.5 and SMIT‐Gβγ puncta per cell in male (black, *N* = 3 *n* = 15), DM female (blue, *N* = 3 *n* = 12) and PE female (red, *N* = 3 *n* = 9) mesenteric (E) and coronary (F) VSMCs. All data is shown as mean ± SEM, where statistical *p* values were calculated using a 2‐way ANOVA and multiple comparisons were made via a post hoc Bonferroni test where *p < 0.05, **p < 0.01, ***p < 0.001, ****p < 0.0001.

## Data Availability

The data that support the findings of this study are availabl in the Materials and Methods, and Results of this article.
